# A multicenter, randomized controlled trial of immediate total-body CT scanning in trauma patients (REACT-2)

**DOI:** 10.1186/1471-227X-12-4

**Published:** 2012-03-30

**Authors:** Joanne C Sierink, Teun Peter Saltzherr, Ludo FM Beenen, Jan SK Luitse, Markus W Hollmann, Johannes B Reitsma, Michael JR Edwards, Joachim Hohmann, Benn JA Beuker, Peter Patka, James W Suliburk, Marcel GW Dijkgraaf, J Carel Goslings

**Affiliations:** 1 Trauma Unit Department of Surgery, Academic Medical Center, Amsterdam, The Netherlands; 2 Department of Radiology, Academic Medical Center, Amsterdam, The Netherlands; 3 Department of Anaesthesiology Academic Medical Center, Amsterdam, The Netherlands; 4 Department of Clinical Epidemiology, Biostatistics and Bioinformatics, Academic Medical Center, Amsterdam, The Netherlands; 5 Trauma Unit Department of Surgery, University Medical Center Sint Radboud, Nijmegen, The Netherlands; 6 Department of Radiology and Nuclear Medicine, University Hospital Basel, Basel, Switzerland; 7 Trauma Unit Department of Surgery, University Medical Center Groningen, Groningen, The Netherlands; 8 Trauma Unit Department of Surgery, Erasmus Medical Center, Rotterdam, The Netherlands; 9 Trauma Unit Department of Surgery, Ben Taub General Hospital, Houston, USA

## Abstract

**Background:**

Computed tomography (CT) scanning has become essential in the early diagnostic phase of trauma care because of its high diagnostic accuracy. The introduction of multi-slice CT scanners and infrastructural improvements made total-body CT scanning technically feasible and its usage is currently becoming common practice in several trauma centers. However, literature provides limited evidence whether immediate total-body CT leads to better clinical outcome then conventional radiographic imaging supplemented with selective CT scanning in trauma patients. The aim of the REACT-2 trial is to determine the value of immediate total-body CT scanning in trauma patients.

**Methods/design:**

The REACT-2 trial is an international, multicenter randomized clinical trial. All participating trauma centers have a multi-slice CT scanner located in the trauma room or at the Emergency Department (ED). All adult, non-pregnant, severely injured trauma patients according to predefined criteria will be included. Patients in whom direct scanning will hamper necessary cardiopulmonary resuscitation or who require an immediate operation because of imminent death (both as judged by the trauma team leader) are excluded. Randomization will be computer assisted. The intervention group will receive a contrast-enhanced total-body CT scan (head to pelvis) during the primary survey. The control group will be evaluated according to local conventional trauma imaging protocols (based on ATLS guidelines) supplemented with selective CT scanning. Primary outcome will be in-hospital mortality. Secondary outcomes are differences in mortality and morbidity during the first year post trauma, several trauma work-up time intervals, radiation exposure, general health and quality of life at 6 and 12 months post trauma and cost-effectiveness.

**Discussion:**

The REACT-2 trial is a multicenter randomized clinical trial that will provide evidence on the value of immediate total-body CT scanning during the primary survey of severely injured trauma patients. If immediate total-body CT scanning is found to be the best imaging strategy in severely injured trauma patients it could replace conventional imaging supplemented with CT in this specific group.

**Trial Registration:**

ClinicalTrials.gov: (NCT01523626).

## Background

Injuries are the cause of 5.8 million deaths annually which accounts for almost 10% of global mortality [1]. Among adults aged 15-59 years the proportion of injuries as cause of death is even higher, ranging from 22% to 29% [1]. Injuries, whether unintentional or intentional, may have devastating effects on the lives of individuals and poses a great burden on public-health budgets [2]. This burden may even increase in the future, since the World Health Organization (WHO) projected a 28% increase in global deaths due to injury between 2004 and 2030 [1].

Specialized trauma centers all over the world provide initial trauma care and diagnostic work-up of trauma patients. This work-up is standardized and frequently based on the Advanced Trauma Life Support (ATLS^®^) guidelines which include a fast and priority-based physical examination as well as screening radiographs supplemented with selective Computed Tomography (CT) [3]. ATLS guidelines advise to routinely perform X-rays of thorax and pelvis and Focused Assessment with Sonography for Tauma (FAST) in trauma patients. X-rays of the spine and extremities are performed based on clinical suspicion during the secondary survey. Whether or not to perform CT scanning following conventional imaging is defined less clearly in the ATLS guidelines and depends upon national guidelines and local protocols.

In recent years CT has become faster, more detailed and more available in the acute trauma care setting. CT shows high accuracy for a wide range of injuries [4-7] which is reflected by a low missed diagnosis rate [5,8-10]. Hence, the conventional radiological work-up according to the ATLS may not be the optimal choice of primary diagnostics anymore. Furthermore, severely injured patients frequently require secondary CT scanning of many parts of the body after conventional imaging. Modern multi-detector CT scanners (MDCT) can perform imaging of the head, cervical spine, chest, abdomen and pelvis in a single examination (total-body CT scanning). The past few years this total-body imaging concept gained popularity as a possible alternative to the conventional imaging strategy. With the use of immediate total-body CT scanning in trauma patients, rapid and detailed information of organ and tissue injury becomes available and a well-founded plan for further therapy can be made.

In the past, CT scanners were located in the radiology department, frequently even on another floor than the emergency department (ED) where the trauma patient is admitted. The past assumption that total-body CT scanning in severely injured trauma patients is too time consuming may no longer be held, since an increasing number of trauma centers have a CT scanner available at the ED or even in the trauma room itself [11,12]. Several studies evaluated time intervals associated with total-body CT usage in severely injured patients [4,5,8,13-18]. Time intervals focused on are scanning time, time to all diagnosis known and time in the ED. Some studies compare different scanning protocols [19-21], some evaluate the effects of a total-body CT scan in one group trauma patients [5,8,9], while others make a comparison in two cohorts trauma patients, one evaluated with an immediate total-body CT scan and one evaluated with ATLS based imaging protocols and selective CT scanning [22-25]. Although these studies are incomparable with respect to design, CT scanners used, diagnostic work-up protocols and trauma populations[26], the main conclusion is clear. Total-body CT scanning in trauma patients is not as time consuming as was once expected and may even be time saving compared to conventional imaging protocols supplemented with selective CT.

The most important question remains whether immediate total-body CT scanning will translate to improved clinical outcome. A recent study in 4621 trauma patients reported a significant increase in the probability of survival for patient given immediate total-body CT scanning compared with conventional imaging strategies supplemented with selective CT scanning [25]. However, since the study was retrospective in nature, no correction for all confounding variables could have been made. Patients who underwent immediate total-body CT scanning were on average more severely injured than those who did not receive total-body CT scanning. Differences between participating centers and protocols used for diagnostic work-up were not described. Whether the positive effect in survival in patients who underwent total-body CT scanning can be attributed solely to the total-body CT scan itself remains therefore unclear.

Although literature provides limited evidence for the usage of an immediate total-body CT scan in the work-up of trauma patients, more and more trauma centers encourage and are implementing immediate total-body CT scanning in the diagnostic phase of primary trauma care. Since the burden of total-body CT scanning in terms of costs and radiation dose is at least controversial [20,27,28], the advantage of performing an immediate total-body CT scan should be proven in high quality studies resulting in high level evidence in order to make its implementation justifiable.

In order to assess the value of immediate total-body CT scanning in severely injured trauma patients, the Academic Medical Center (AMC) in Amsterdam, the Netherlands, has initiated an international multicenter randomized controlled trial. Severely injured patients, who are thought to benefit the most from a total-body imaging concept, will be included. Such a trial has never been done before and is crucial to provide evidence whether or not the usage of immediate total-body CT scanning in the diagnostic phase of primary trauma care is justifiable.

## Methods/design

### Study objectives

The primary objective is to determine the effects of immediate total-body CT scanning during the primary trauma survey on clinical outcomes compared to patients who are evaluated with standard conventional Advanced Trauma Life Support (ATLS^®^) based radiological imaging. The secondary objectives are to assess the effects of total-body CT scanning on long term clinical outcomes, quality of life, clinically relevant time intervals in the early phase of trauma care and the differences in treatment strategies used.

### Study design

The REACT-2 trial is an international, multicenter randomized clinical trial in six high-volume trauma centers that will compare the effects of immediate total-body CT scanning in severely injured trauma patients with conventional imaging protocols.

### Setting

In total four trauma centers in The Netherlands, one Swiss and one American trauma center will participate in the REACT-2 trial. All participating hospitals are level-1 trauma centers with a multi-slice CT scanner located in the trauma resuscitation room or at the ED.

**Figure 1:**
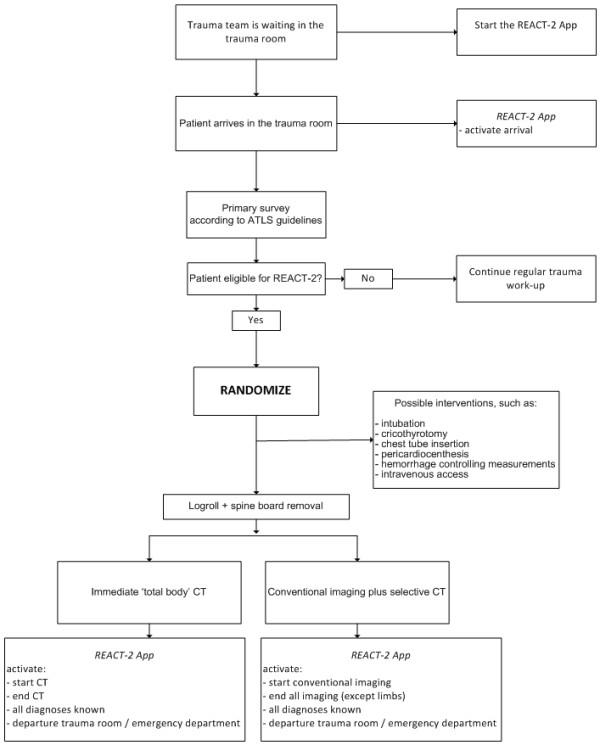
**Study flow chart REACT-2 trial**.

When a patient arrives in the trauma room a brief report of the pre-hospital circumstances, medical assessment and clinically suspected injuries is presented to the trauma team leader by the ambulance personnel. The initial evaluation of trauma patients will be done according to the ATLS guidelines for the primary survey. Potential life-saving interventions during the primary survey and before any imaging include securing the airway by intubation or performing a cricothyrotomy, chest tube insertion, pericardiocenthesis or taking hemorrhage controlling measurements such as applying a pelvic binder or external pressure on bleeding sites to (temporarily) stabilize the vital functions. Usually, peripheral intravenous access is taken care of by the ambulance personnel, but if not, at least one intravenous catheter will be inserted before radiologic imaging takes place. Based on information received from the ambulance personnel and the findings during primary survey, the trauma team leader decides on the eligibility of the patient to participate in the trial. If the patient is found to be eligible randomization takes place. Figure 1 depicts the study flow chart.

The intervention group will receive a total-body CT scan from head to pelvis. In the intervention group conventional radiography of the torso and FAST will be completely omitted. The CT protocol for the consists of a two-step whole-body acquisition (from vertex to pubic symphysis) starting with Head and Neck Non Enhanced CT (NECT) with arms alongside the body. The preferred technique for the second complementary scan is a split-bolus intravenous contrast directly after repositioning of the arms alongside the head, and this second scan covers thorax, abdomen and pelvis. Participating centers however are free to choose their own technique as long as intravenous contrast is given for the chest and abdominal part of the total-body CT.

The control group will be evaluated according to a conventional trauma protocol with selective CT scanning. The REACT-2 trial has been designed to maximize the applicability of the trial's results to usual care settings. Therefore, the technical details of the CT scanning done in the control group are not specified and participating centres follow their own protocols. Indications for the selective CT scanning however are pre-defined based on the combined local protocols of the participating centers. These standardized protocols provide a basis for the comparison of the two imaging approaches.

### Study population

All non-pregnant trauma patients aged 18 years and older having life-threatening (respiratory, circulatory or neurologically) conditions with compromising vital parameters, with clinical suspicion on specific injuries or with specific injury mechanisms are included. Patients in whom the scanning will hamper necessary (cardiopulmonary) resuscitation or who require an immediate operation because of imminent death (both as judged by the trauma team leader) are excluded. Detailed in- and exclusion criteria are summarized below:

### Inclusion criteria

Trauma patients with the presence of life-threatening vital problems defined as at least one of the following:

- respiratory rate ≥ 30 min of ≤ 10/min;

- pulse ≥ 120/min;

- systolic blood pressure ≤ 100 mmHg;

- estimated exterior blood loss ≥ 500 ml;

- Glasgow Coma Score ≤ 13;

- Abnormal pupillary reaction onsite.

OR

Patients with one of the following clinically suspicious diagnoses:

- flail chest, open chest or multiple rib fractures;

- severe abdominal injury;

- pelvic fracture;

- unstable vertebral fractures/spinal cord compression;

- fractures from at least two long bones.

OR

Patients with one of the following injury mechanisms:

- fall from height (> 3 m/> 10 ft);

- ejection from the vehicle;

- death occupant in same vehicle;

- severely injured patient in same vehicle;

- wedged or trapped chest/abdomen.

### Exclusion criteria

Trauma patients with one of the following characteristics will be excluded:

- known age < 18 years;

- known pregnancy;

- referred from another hospital;

- clearly low-energy trauma with blunt injury mechanism;

- penetrating injury in 1 body region (except gun shot wounds) as the clearly isolated injury;

- any patient who is judged to be too unstable to undergo a CT scan and requires (cardiopulmonary) resuscitation or immediate operation because death is imminent according to the trauma team leader in mutual agreement with the other leading care givers.

### Endpoints

The primary outcome criterion for this trial is in-hospital mortality.

As secondary parameters for the trial focus on additional clinical consequences for the patients and cost-effectiveness and cost-utility:

- mortality (24-h, 30-day and 1-year mortality);

- morbidity (complications and total number of (re-)interventions and re-admissions up to 6 months post trauma; transfusion requirements, length of ICU stay and number of ventilation days);

- several time intervals during initial evaluation (time of arrival, time to CT, scanning time, time to diagnosis and time in the ED);

- radiation exposure;

- quality of life 6 and 12 months after the trauma as recorded by completing the EuroQol-6D;

- general health 6 and 12 months after the trauma as recorded by completing the HUI-3;

Economic parameters/endpoints:

- total costs of imaging during the initial/index hospital stay;

- total direct and indirect medical and non-medical costs during the first half year posttrauma;

- quality adjusted life-years (QALY's).

### Randomization

If a patient is eligible for the trial the diagnostic imaging pathway for initial assessment in the trauma resuscitation room will be determined by randomization. The randomization will be performed immediately after inclusion at computers located in the trauma room of the participating hospitals. Randomization will be performed using a 'one-click' computer program on a 1:1 basis per hospital with varying block sizes of 2, 4, 6, 8, 10 and 12. The trauma team will be directly informed on the outcome of the randomization so that imaging can be started. A standardized case record from (CRF) will be used. This CRF is totally web-based via a secured internet module.

### Sample size calculation and data analysis

A previous study reported a reduction in mortality from 15% to 8.6% with total-body CT scanning as the single diagnostic procedure during trauma evaluation as compared to historical control data [29]. Analysis on the large German polytrauma registration database performed by Huber-Wagner et al. showed a significant reduction in the 24-h mortality in patient who underwent immediate total-body CT compared to the conventional group (10% vs. 12%, P = 0.038) [25]. Historical AMC data show a mortality rate of 12% for trauma patients matching the current trial inclusion criteria. Based on the combination of the AMC data and the participation of the other trauma centers with comparable trauma populations, it is expected to find a reduction in mortality from 12% to 7%. The detection of such a difference requires 539 patients per group using a power of 80% and a two-sided alpha of 5%. Based on the historical and estimated data of the participating centers the inclusion period will take about 1,5 years and the follow-up period will take an additional year.

The main analyses of primary and secondary outcomes will be conducted for all randomized patients according to the result of the randomization (intention-to-treat). Data are expressed as percentages for categorical data, as mean and standard deviation (SD) for normally distributed numerical data and as median, range, and, where appropriate, inter-quartile range (IQR = 25 to 75%) for non-normally distributed numerical data.

The following subgroups will be used for subgroup analysis:

- multitrauma patients (defined as Injury Severity Score (ISS) >/=16);

- severe traumatic brain injury patients (defined as admission Glasgow Coma; Scale (GCS) ≤ 8 and an Abbreviated Injury Score (AIS)-head of ≥ 3);

- penetrating versus blunt trauma.

A p-value less than 0.05 is considered statistically significant. If appropriate, predictive values between variables are calculated. Predictive values in continuous outcome variables are assessed using a multivariate regression model, and binary outcome measures are assessed using a multivariate logistic regression model. In case of binary outcome measures, predictive values are expressed as Odds Ratio's (OR) with 95% Confidence Intervals (CI). Data are analyzed using the Statistical Package for the Social Sciences (SPSS) version 18.0 SPSS Inc., Chicago, IL.

### Economic evaluation and cost analysis

Total-body CT scanning will be evaluated economically from a societal perspective against a conventional diagnostic strategy consisting of X-ray, FAST and selective CT scanning according to the ATLS guidelines. Cost-effectiveness analyses will be performed with the costs per patient alive and costs per patient alive without serious morbidity as outcome measures. Additionally, a cost-utility analysis will be done with the cost per QALY as outcome measure. Incremental cost-effectiveness ratios will be calculated, expressing the extra costs per (i) extra patients alive, (ii) extra patients alive and without serious morbidity, and (iii) additional QALY. Sampling variability will be accounted for by (bias-corrected and accelerated) non-parametric bootstrapping. Sensitivity analyses will be directed at applied QALY algorithms (generic, country-specific; uniform, linear, curvilinear interpolations between measurements), unit costs of major cost components, and the (country-specific) friction period in case of production loss. Subgroup analyses will be performed by the predefined subgroups. The time horizon for the cost-effectiveness analysis will be six months following trauma. Because of this time horizon, no discounting will take place.

The economic evaluation will take all direct and indirect medical and non-medical costs into account. The direct and indirect medical costs include the costs of initial trauma care, ICU-care and care at the general ward during the index admission - including all diagnostic and therapeutic procedures - as well as the costs of repeat hospital admissions, other intramural care like rehabilitation and extramural care during the first 6 months post trauma. Direct and indirect non-medical costs of, respectively, out-of-pocket expenses and production loss during the first 6 months will also be estimated. Volume data will be collected by case report form, institutional administrative databases and by patient questionnaires at 3 and 6 months, depending on the cost category. The patient questionnaire will be derived from the Dutch Health and Labour Questionnaire and adapted for international use. Unit costing will be based on activity based costing and hospital ledger data concerning the major diagnostic procedures in this trial. Unit costing of other health care components will be based on available national guidelines. In case of absence of national guidelines in specific countries, available unit costs from abroad will be recalculated using Organisation for Economic Co-operation and Development (OECD) purchasing power parities. Out-of-pocket expenses will be estimated as supplied by the patients. Indirect costs of production loss will be calculated according to the Dutch perspective by following the friction cost method, while applying the most recent friction cost period known at the time of analysis. Costs will be calculated for the base year 2012. Unit costs of other base years will be price-indexed.

### Safety monitoring

An independent Data and Safety Monitoring Board (DSMB), consisting of three members (2 physicians and 1 clinical epidemiologist), is installed for this trial. On regular intervals, this committee will review accumulating trial data and provide advice on the conduct of the trial to the trial leader and Steering Committee. The DSMB will focus both on safety and effectiveness data. Standard Operating Procedures (SOP) will be used with respect to the schedule and format of DSMB meetings and with respect to the format and timing of presenting data. The DSMB can recommend the Steering Committee to terminate the trial when there is clear and substantial evidence of harm.

#### Safety and efficacy monitoring

The role of the DSMB is to perform an interim review of the trial's progress including updated figures on main outcomes and safety data. This review would include, but not be restricted to, the following:

• monitor compliance with the protocol by participants and investigators;

• monitor evidence for treatment differences in the main efficacy outcome measures;

• monitor evidence for treatment harm (e.g. SAEs, deaths);

• decide whether to recommend that the trial continues to recruit participants or whether recruitment should be terminated either for everyone or for some treatment groups and/or some participant subgroups;

• suggest additional data analyses;

• monitor compliance with previous DSMB recommendations;

• consider the ethical implications of any recommendations made by the DSMB;

• assess the impact and relevance of external evidence as supplied by the Chief Investigator.

The DSMB will evaluate these safety and efficacy parameters at regular intervals. After 275 (25%), 550 (50%) and 700 (65%) included patients, non-blinded interim-analyses for evaluation of safety rules will be performed. No formal stopping rules based on statistical criteria alone will be used. The DSMB decides after evaluation of all necessary interim data whether the trial will be continued or terminated. Other investigators, designated by the Board of Direct of the AMC to control the trial will have the authority to gain insight in all the confidential data relevant for the trial as well.

### Ethics

This trial is conducted in accordance with the principles of the Declaration of Helsinki [30], the Medical Research Involving Human Subjects Act (WMO) and 'Good Clinical Practice' guidelines. The Medical Ethical Committee of the Academic Medical Center in Amsterdam has approved the protocol on January 6 2011. The Ethical Committees of the participating centers approved for local feasibility.

To participate in a research project the subjects must be volunteers and informed participants according to ethical principles stated in the Declaration of Helsinki. However, the acute life-threatening situation of severely injured trauma patients hinders a considered decision. Neither a legal guardian nor a legal representative of the patient can make a decision because of the time pressure or because they simply do not arrive in time. A temporary waiver of informed consent during randomization and the consecutive diagnostic phase during trauma survey was approved by the Medical Ethical Committee of the Academic Medical Center in Amsterdam. In all cases informed consent will be asked afterwards from the patient or the legal guardian/representative of the patient, as soon as reasonably possible.

## Discussion

The need for prospective studies to measure the effect of immediate total-body CT scanning in trauma care has been stressed recently by several authors [8,22,23,25,29]. Retrospective studies have shown the possible benefits in time and outcome of immediate total-body CT scanning in trauma patients. The next step is to compare its usage to the current best imaging strategy according to ATLS guidelines in a prospective trial.

The primary question that needs to be answered is whether immediate total-body CT scanning in severely injured trauma patients decreases mortality and significant morbidity when compared to conventional imaging strategies supplemented with CT. Therefore, randomization is within the hospital, ensuring that a comparison between imaging protocols is made per hospital instead of between hospitals. The design of the trial is multi-centered, with participating centers in The Netherlands, Switzerland and North America. This design assures that differences in trauma populations, trauma mechanisms and workflow in different parts of the world are taken into account as well. This is important to make sure that if an effect on outcome is seen that this can solely be attributed to the usage of a total-body CT scan.

The in- and exclusion criteria assure that only potentially severely injured trauma patients are included and over triage is minimized. Especially severely injured patients are thought to benefit the most from fast and detailed information that becomes available with total-body CT scanning. Selecting the right patients for immediate total-body CT scanning is therefore crucial. Since the excluded trauma patients will be registered as well, final analysis will show whether the chosen inclusion criteria led to an appropriate selection of patients. Furthermore, severely injured patients are those patients in whom the radiation dose may be justifiable since their possible life-threatening injuries require accurate treatment as fast as possible. Trauma patients are exposed to a great amount of radiation and it is well known that CT scanning is a significant contributor to iatrogenic radiation exposure [31]. The mean effective dose received by trauma patients evaluated by conventional imaging protocols supplemented with CT scanning was found to be 22.7 milliSievert (mSv) [32]. A single total-body CT scan accounts for 14-21 milliGray (mGy), which in medical X-ray studies is equal to mSv [31]. However, cumulative doses for all the radiological examinations undertaken during hospitalization may be much higher [33]. The long-term effects of the radiation exposure are based upon estimations, but the most concerning is an increased cancer risk. For a single total-body CT examination the estimated lifetime attributable cancer mortality risk is thought to be around 0.08% [31].

After conventional imaging in terms of X-rays and ultrasound has been finished the trauma leader has to decide whether or not selective CT should take place. The ATLS guidelines provide some decision rules but to some extent it is susceptible to individual judgment. Experience of the trauma leader and local infrastructures may influence these decisions. Furthermore, the randomization between total-body CT and conventional imaging supplemented with CT within each center holds the risk of a learning curve experienced by trauma leaders. If the trauma leader suspects detecting more injuries with a total-body CT scan than was expected on clinical grounds, performing selective CT scanning in the conventional arm could become more easily accessible and may lower the possible differences in outcome between the study groups. That is why the indication for selective CT scanning in the conventional arm are pre-defined, based on combined local protocols of the participating centers. The standardization of the conventional arm will lower the aforementioned risks.

This trial aims to determine the optimal diagnostic strategy for severely injured trauma patients in the ED. If immediate total-body CT scanning is found to be the best imaging strategy in severely injured trauma patients it could replace conventional imaging supplemented with CT in this specific group. This will probably minimize the total diagnostic work-up time of the initial trauma evaluation. How this reflects in outcome needs to be analyzed in this trial. Furthermore, severely injured patients are already likely to receive selective CT scanning after conventional imaging according to ATLS guidelines or according to local trauma protocols. Segmented CT scanning in these patients, added to the conventional work-up, will result in a high total radiation dose because of overlapping radiation fields. It could therefore even be possible that an immediate total-body CT results in a lower the total effective radiation dose compared to the conventional work-up with selective CT scanning [27].

The trial not only focuses on clinical outcome in terms of mortality and morbidity. Since radiation exposure and cost-effectiveness will be taken into account as well, the REACT-2 trial will provide a detailed overview of considerations that should be taken into account when discussing the efficacy of immediate total-body CT scanning in trauma patients. The large sample size will make sure that results are reliable and can be generalized to all international trauma populations and centers.

## Conclusion

The REACT-2 trial is an international multicenter randomized clinical trial http://ClinicalTrials.gov/NCT01523626 to compare immediate total-body CT scanning during the primary survey of severely injured trauma patients with conventional imaging strategies supplemented by selective CT scanning.

### Prospective

The REACT-2 inclusion has started in April 2011. Results are expected in mid 2014.

## Abbreviations

ATLS: Advanced Trauma Life Support; AIS: Abbreviated Injury Score; AMC: Academic Medical Center; ED: Emergency Department; FAST: Focused Assessment with Sonography for Trauma; GCS: Glascow Coma Scale; ICU: Intensive Care Unit; mGy: Milligray; ISS: Injury Severity Score; mSv: Millisievert; REACT-2: Randomized study of Early Assessment by CT scanning in Trauma patients -2; CT: Computed Tomography.

## Competing interests

J.C. Sierink, MD, is a Ph.D.-student at the Trauma Unit Department of Surgery, employed by the AMC Medical Research B.V., and supported by an unrestricted grant from ZonMw, the Netherlands organisation for health research and development (grant number: 1711020323). All authors declare that they have no competing interests.

## Authors' contributions

JCS drafted the manuscript, TPS and JCG co-authored the writing of the manuscript. All authors participated actively in the design of the trial and critically appraised the manuscript. All authors read and approved the final manuscript.

## Pre-publication history

The pre-publication history for this paper can be accessed here:

http://www.biomedcentral.com/1471-227X/12/4/prepub
